# The impact of large and small dams on malaria transmission in four basins in Africa

**DOI:** 10.1038/s41598-021-92924-3

**Published:** 2021-06-25

**Authors:** Solomon Kibret, Matthew McCartney, Jonathan Lautze, Luxon Nhamo, Guiyun Yan

**Affiliations:** 1 Program in Public Health, University of California Irvine, Irvine, CA 92697 USA; 2 International Water Management Institute, Colombo, Sri Lanka; 3 International Water Management Institute, Pretoria, South Africa; 4 Present Address: Water Research Commission, Pretoria, South Africa

**Keywords:** Ecology, Diseases, Environmental impact

## Abstract

Expansion of various types of water infrastructure is critical to water security in Africa. To date, analysis of adverse disease impacts has focused mainly on large dams. The aim of this study was to examine the effect of both small and large dams on malaria in four river basins in sub-Saharan Africa (i.e., the Limpopo, Omo-Turkana, Volta and Zambezi river basins). The European Commission’s Joint Research Center (JRC) Yearly Water Classification History v1.0 data set was used to identify water bodies in each of the basins. Annual malaria incidence data were obtained from the Malaria Atlas Project (MAP) database for the years 2000, 2005, 2010 and 2015. A total of 4907 small dams and 258 large dams in the four basins, with 14.7million people living close (< 5 km) to their reservoirs in 2015, were analysed. The annual number of malaria cases attributable to dams of either size across the four basins was 0.9–1.7 million depending on the year, of which between 77 and 85% was due to small dams. The majority of these cases occur in areas of stable transmission. Malaria incidence per kilometre of reservoir shoreline varied between years but for small dams was typically 2–7 times greater than that for large dams in the same basin. Between 2000 and 2015, the annual malaria incidence showed a broadly declining trend for both large and small dam reservoirs in areas of stable transmission in all four basins. In conclusion, the malaria impact of dams is far greater than previously recognized. Small and large dams represent hotspots of malaria transmission and, as such, should be a critical focus of future disease control efforts.

## Introduction

### Dams are key for development

To ensure food security and promote resilience, African governments have embarked on major programs to expand the continent’s water infrastructure over the past 2 decades^1^. Many large and small dams, as well as household-level storage structures, are currently under construction, particularly in sub-Saharan Africa (SSA), a region where infrastructure development lags behind other developing countries and where per capita water storage is amongst the lowest in the world^2^. Unintended impacts of such infrastructure on vector borne diseases, such as malaria, continue to be a pressing public health challenge in this region^3^.

### Impact of large vs. small dams on malaria

The impact of large dams (i.e., dams with a height of 15 m or greater from lowest foundation to crest or a dam between 5 and 15 m impounding more than 3 million cubic meters (m^3^) of water) on malaria has been widely documented around individual reservoirs in different parts of Africa^4–11^. The aggregate effect of large dams on malaria in sub-Saharan Africa (SSA) has also been assessed^12^. The results provided a conservative estimate that at least 1.1 million malaria cases annually can be attributed to the presence of large dams in SSA. In contrast, with the exception of a few individual dams in the Tigray region of Ethiopia^13^ and in Burkina Faso^14^, the impact of small dams (i.e., dams creating reservoirs with surface area less than 100 hectares (ha) or with a storage capacity below 3 million m^3^ behind a dam that is less than 15 m high) has not been extensively investigated. In the Tigray region, a seven-fold increase in malaria transmission was found in the vicinity of small dams compared to villages where no dams had been built^13^. Evidence from Burkina Faso was less conclusive with no significant impact of small dams observed^14^. No previous studies have examined the aggregated malaria impacts of small dams, nor compared the aggregated malaria impact of small dams with that of large dams, over a specific geography. Generation of knowledge on the relative impact of small and large dams on malaria could help to inform the future direction of water planning and management.

The study reported here, compared the effect of small and large dams on malaria in SSA using an approach based on precise reservoir delineation. The study focused on four basins, Limpopo, Omo-Turkana, Volta, and Zambezi, which cumulatively contained 258 and 4907 georeferenced large and small dams, respectively, in 2015. We first describe how reservoirs were delineated using remotely-sensed perimeters. The effects of small and large dams on malaria were then determined in each basin. Malaria transmission in communities close to (< 5 km) and far from (5–10 km) the small and large reservoirs were compared and implications for malaria control deduced.

## Methods

### Study area

**Figure 1:**
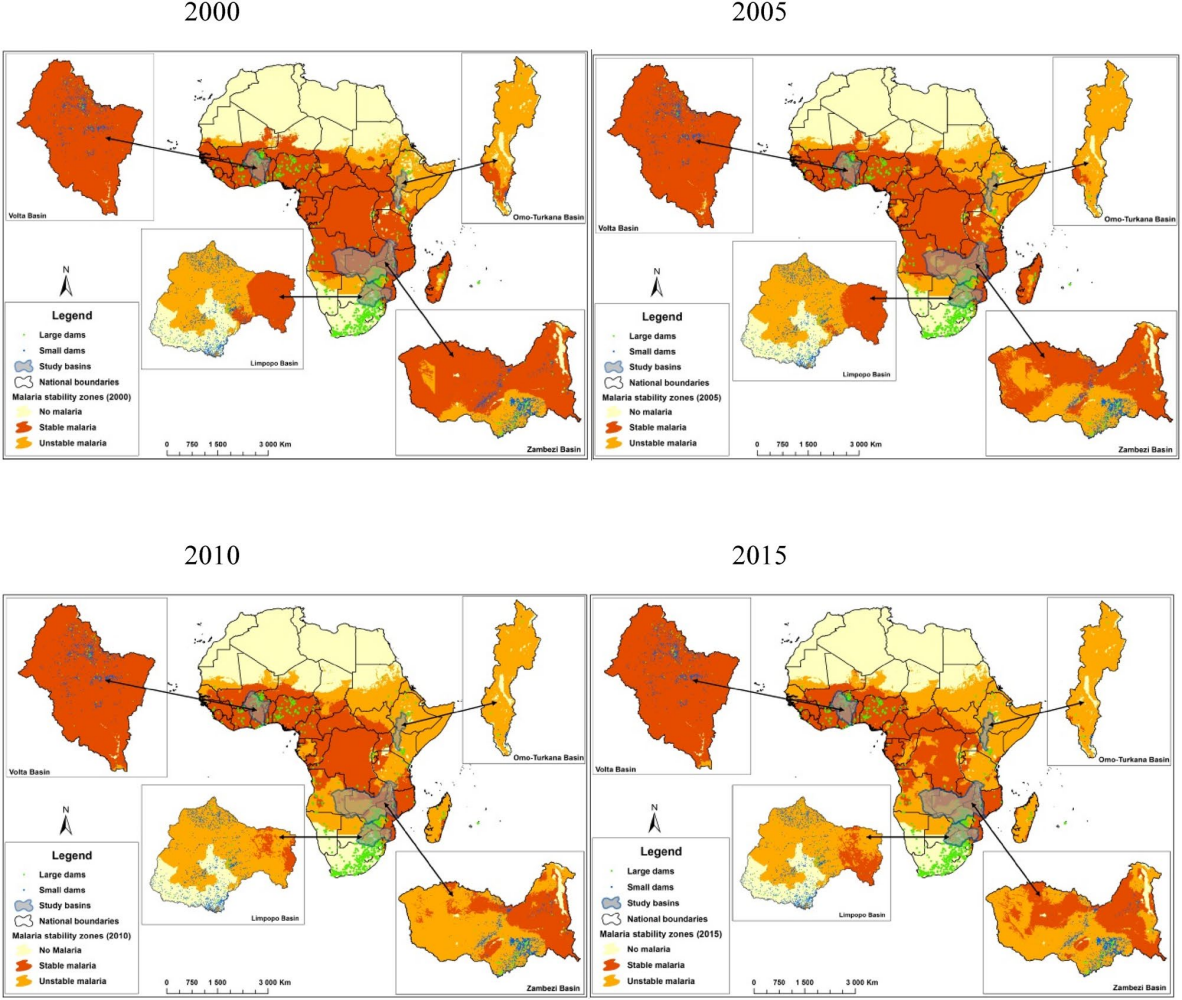
Distribution of large and small dams in Limpopo, Volta, Zambezi and Omo-Turkana basins by malaria stability zone. [The figure was made using open- source malaria data^23^ on ArcGIS software (version 10.5. 1, Environmental Systems Research Institute Inc, Redlands, CA, USA, 2016)].

Four major river basins, located across different sub-regions of SSA, were selected for this study: Limpopo, Omo-Turkana, Volta, and Zambezi (Fig. 1). These basins were selected to (i) foster inclusion of enable different African regions and (ii) ensure focus on basins with sufficient data availability.

The Limpopo River basin is located in southern Africa. Draining an area of approximately 408,000 km^2^, the Limpopo River basin is distributed among South Africa (45%), Botswana (20%), Zimbabwe (15%) and Mozambique (20%). About 14 million people live in this basin. The climate of the Limpopo River basin varies along the path of the river from a temperate climate in the west to a subtropical climate at the river mouth in Mozambique. The hydrology of the Limpopo River basin is influenced by the highly seasonal distribution of rainfall over the catchment. About 95% of rain falls between October and April with a peak normally in February. Temperature varies from 30 to 34 °C in summer and 22–26 °C in winter^15^.

The Volta River basin is located in West Africa with a population of over 23 million. Draining an area of 409,000 km^2^ the basin is spread across six countries: Benin (4%), Burkina Faso (42%), Cote d’Ivoire (3%), Ghana (41%), Mali (4%) and Togo (6%). Average annual rainfall varies across the basin from approximately 1600 mm in the southeast, to about 360 mm in the north. Annual mean temperatures in the basin vary from 27 to 30 °C^16^. The main rainy season is between March and October.

The Zambezi River basin is located in southern Africa. Draining an area of 1.34 million km^2^, the basin is spread across eight countries: Angola (19%), Botswana (1%), Namibia (1%) Benin (4%), Zimbabwe (16%), Zambia (42%), Tanzania (2%), Malawi (8%) and Mozambique (12%). The population of the Zambezi basin is estimated to be about 32 million. Annual rainfall in the basin ranges from 550 mm in the south to 1800 mm in the north. The annual mean temperatures ranges from 18 °C at higher elevations in the south of the basin to 26 °C for low elevations in the delta in Mozambique^17^.

The Omo-Turkana Basin covers approximately 131,000 km^2^, stretching from southern Ethiopia to northern Kenya. Hydrologically, the basin is dominated by Lake Turkana, with the Omo River, which drains the Ethiopian portion of the basin, supplying 90% of the inflow to the lake. The basin is home to approximately 15 million people, the majority of whom live in the Ethiopian highlands, in the north. The annual mean temperature ranges from 24 °C in the north to 29 °C in the south. The mean annual rainfall ranges from 250 mm in the south to 500 mm in the north^18^.

### Data sources

#### Dam data

##### Small dams

Data on location and size of small dams are not readily available in either global or regional data sets. The European Commission’s Joint Research Center (JRC) Yearly Water Classification History v1.0 data set was used to identify water bodies in each of the four basins^19^. Water bodies less than 100 ha and greater than 2 ha were identified. All were checked with Google Earth images to distinguish between reservoirs and natural water bodies (Supplementary Fig. S1). Ultimately, a total of 4907 small dams located in the four basins were identified and included in the analyses.

##### Large dams

For large dams, the FAO African Dams Database^20^, International Commission for Large dams (ICOLD)^21^ and the International Rivers Database^22^, which together contain 1286 georeferenced African large dams, were utilized. The accuracy of dam locations was first verified with Google Earth. When the location of a dam did not precisely match the coordinates stipulated in either of the two databases, manual corrections were made by adjusting the coordinates of a dam to its location as shown in Google Earth (see Supplementary Information). Dams for which precise locations could not be determined, as well as dams without reservoirs (i.e., run-of-river schemes), were removed. Ultimately, across the four basins, a total of 258 large dams with confirmed georeferenced locations were identified and included in the analyses.

##### Perimeters of large and small dam reservoirs

Reservoir perimeters of both large and small dams were extracted from the European Commission’s Joint Research Center (JRC) global surface water datasets^19^, published through the Google Earth Engine. This dataset includes maps of the location and temporal variability in maximum perimeter records of the global surface water coverage from 1984 to 2015. In this study, the maximum perimeter records were used in each year of 2000, 2005, 2010 and 2015. The data were exported to ArcGIS.

##### Data on anopheles mosquito distribution

Data for vector distribution were obtained from the Malaria Atlas Project (MAP) database^23^. The MAP database contains a georeferenced illustration of the major malaria vector species in different malaria-endemic areas in Africa.

##### Malaria data

Annual malaria incidence data were obtained from the MAP database. We acquired data for the years 2000, 2005, 2010 and 2015. These years were selected to align with updates to Worldpop population data^24^, which are recomputed every five years. MAP produced a 1 km resolution continuous map of annual malaria incidence for Africa based on 33,761 studies across the region. We imported these data to ArcGIS for analyses. Annual malaria incidence was determined as the number of cases per 1000 population. To ascertain the impact of dams on malaria incidence rates as a function of distance from the reservoir perimeter, we created two distance zones: 0–5 km (at risk) and 5–10 km (control). When distance zones were overlapping for two or more nearby dams, areas were assigned to the closest distance cohort. Populations residing more than 5 km from a reservoir perimeter (large or small) were considered to be free of risk from dam induced malaria transmission because the maximum mosquitoes’ flight range is considered to be < 5 km^25^. Hence, the 5–10 km zone served as a control.

##### Population data

Annual population data of SSA were obtained from the Worldpop database^24^. A 1 × 1 km gridded population map was imported to ArcGIS for analyses. The total number of people living in each distance cohort was determined for each reservoir every 5 years for the period 2000–2015.

### Data analyses

#### Mapping vector distribution around small and large dams

To illustrate the locations of reservoirs with respect to different *Anopheles* species, we superimposed malaria vector distribution obtained from the MAP database on the small and large dams in the four basins to show the risk of malaria transmission around reservoirs in areas with different vector compositions.

#### Distribution of small and large dams in areas of different stability

Different studies use different approaches to describe malaria stability^26,27^. We followed Gething et al.^26^ where areas were categorized as stable (> 0.1 malaria cases per 1000 population), unstable (≤ 0.1 malaria cases per 1000 population) and no malaria (zero malaria incidence) based on the level of malaria incidence in each of the four years: 2000, 2005, 2010, and 2015. The number of dams in each of the three stability categories for each of the four years was determined, as well as the population at-risk of dam-related malaria (i.e., < 5 km from reservoir shorelines).

#### Malaria incidence around small and large dams

The number of annual malaria cases was estimated for the two distance cohorts (< 5 km and 5–10 km) by multiplying malaria incidence rates by the population in each zone. Repeated analysis of variance (ANOVA) was applied to determine differences in malaria incidence between the two cohorts, followed by post hoc HSD Tukey’s test^28^.

#### Incidence per km of reservoir shoreline

Entomological investigation of dam-associated malaria transmission^29^ suggests that reservoir shoreline constitutes the most important breeding habitat and malaria risk factor in dam-affected geographies. As such, comparison of the relative malaria impact of small and large dams was determined by computing the average number of malaria cases per km of reservoir shoreline. This was calculated for each reservoir by dividing malaria incidence in the < 5 km cohort by the reservoir perimeter. The average incidence per km was computed separately for small and large reservoirs in each basin for each of the four years 2000, 2005, 2010 and 2015.

#### Malaria cases attributable to dams

The annual number of malaria cases attributed to dams was determined for areas of both unstable and stable transmission. To do so, the rate of transmission in communities 5–10 km from reservoirs to the population living within 5 km of reservoirs was applied to gauge the number of malaria cases that could be presumed to occur in the absence of the dam. This was calculated as (I_1_–I_2_) P, where I_1_ is malaria incidence in communities living within 5 km of reservoirs, I_2_ is malaria incidence in communities living between 5 and 10 km from reservoirs, and P is the total human population in communities living within 5 km of reservoirs.

## Results

### Dams locations in relation to malaria stability

By 2015, the entire Volta basin was located in a region of stable malaria transmission while the Limpopo, Omo-Turkana and Zambezi were mainly in regions of unstable transmission (Fig. 1). In 2000, the Omo-Turkana basin was largely unstable, whereas notable portions of Limpopo and Zambezi were in areas of stable transmission. By 2015, minimal areas in the Limpopo and Zambezi basins were located in regions of stable malaria transmission.

### Total number of small and large dams in the four basins

The aggregate number of small dams far exceeds the number of large dams. A total of 4907 small dams and 258 large dams existed in 2015 in the Limpopo, Omo-Turkana Volta, and Zambezi basins (Table 1). Over the 15 years’ study period, the total number of small and large dams has not changed substantially. The Zambezi basin possesses the highest total number of geo-located dams (i.e. 73 large and 2566 small in 2015), whereas the Omo-Turkana basin has the smallest total (i.e. 6 large and 41 small in 2015) (Table 1).

**Table 1:** Summary of number of dams in Limpopo, Omo-Turkana, Volta and Zambezi basins.

Year	Volta		Limpopo		Zambezi		Omo-Turkana	
	No. of small dams	No. of large dams	No. of small dams	No. of large dams	No. of small dams	No. of large dams	No. of small dams	No. of large dams
2000	702	55	1549	123	2448	72	36	3
2005	708	55	1552	124	2496	73	39	4
2010	712	55	1575	124	2526	73	40	5
2015	717	55	1583	124	2566	73	41	6

### Predominant Vectors around dams in the four basins

**Figure 2:**
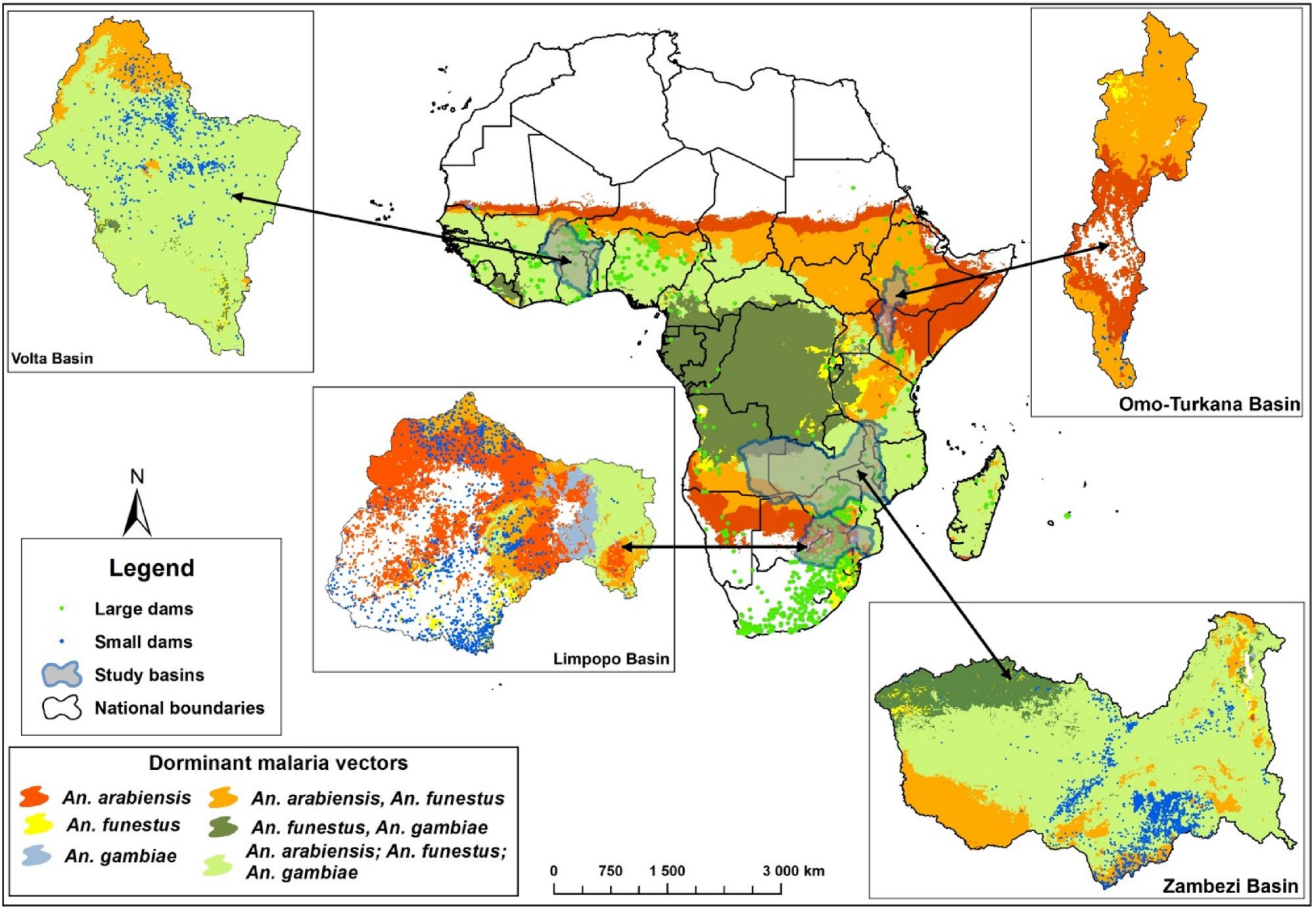
Malaria vector species distribution in Limpopo, Volta, Zambezi and Omo-Turkana basins. [The map was developed by superimposing the MAP data ^29^ on shapefiles of the basins on ArcGIS software (version 10.5. 1, Environmental Systems Research Institute Inc, Redlands, CA, USA, 2016)].

The three major malaria vector species *An. gambaie*, *An. funestus* and *An. arabiensis* were commonly spread around the Volta and Zambezi basins (Fig. 2). The Limpopo and Omo-Turkana possess a more fragmented distribution of vector species with a different predominant vector in different portions of the basins. Typically, *An. arabiensis* predominated in the Limpopo and Omo-Turkana basins while *An. gambiae, An. arabiensis* and *An. funestus* co-existed in the Volta and Zambezi basins.

### Population at-risk around dams in the four basins

A total of 14.7 million people lived close (< 5 km) to reservoirs (small and large dams) in the four basins in 2015; the majority (12.3 million) lived in the vicinity of small dam reservoirs (Table 2; Table 3). The Zambezi River basin accounted for over a third of the population living close to dams in the four basins.

**Table 2:** Summary of cumulative reservoir shoreline and population living close to small dam reservoirs in the Volta, Limpopo, Zambezi and Omo-Turkana basins.

Basins	Year							
	2000		2005		2010		2015	
	Total reservoir shoreline (km)	Population < 5 km from shoreline	Total reservoir shoreline (km)	Population < 5 km from shoreline	Total reservoir shoreline (km)	Population < 5 km from shoreline	Total reservoir shoreline (km)	Population < 5 km from shoreline
Volta	1801	2,422,651	1819	2,816,103	1836	3,289,095	1852	4,170,071
Limpopo	2065	1,077,956	2065	1,270,306	2065	1,523,519	2105	1,844,046
Zambezi	4767	3,732,630	4777	3,857, 963	4795	4,106,999	4823	6,116,575
Omo-Turkana	53	60,512	57	81,311	59	104,831	60	126,287
Grand total	8686	7,286,749	8718	8,025,683	8755	9,024,444	8840	12,256,979

**Table 3:** Summary of cumulative reservoir shoreline and population living close to large dam reservoirs in the Volta, Limpopo, Zambezi and Omo-Turkana basins.

Basins	Year							
	2000		2005		2010		2015	
	Total reservoir shoreline (km)	Population < 5 km from shoreline	Total reservoir shoreline (km)	Population < 5 km from shoreline	Total reservoir shoreline(km)	Population < 5 km from shoreline	Total reservoir shoreline (km)	Population < 5 km from shoreline
Volta	19,640	383,272	19,640	545,345	19,640	643,072	19,640	815,864
Limpopo	2158	203,518	2158	235,321	2158	274,071	2158	367,364
Zambezi	12,985	325,420	12,985	573,374	12,985	778,228	12,985	903,153
Omo-Turkana	190	132,074	200	172,344	295	193,273	409	408,713
Grand total	34,973	1,044,283	34,983	1,526,385	35,078	1,888,643	35,192	2,495,094

**Figure 3:**
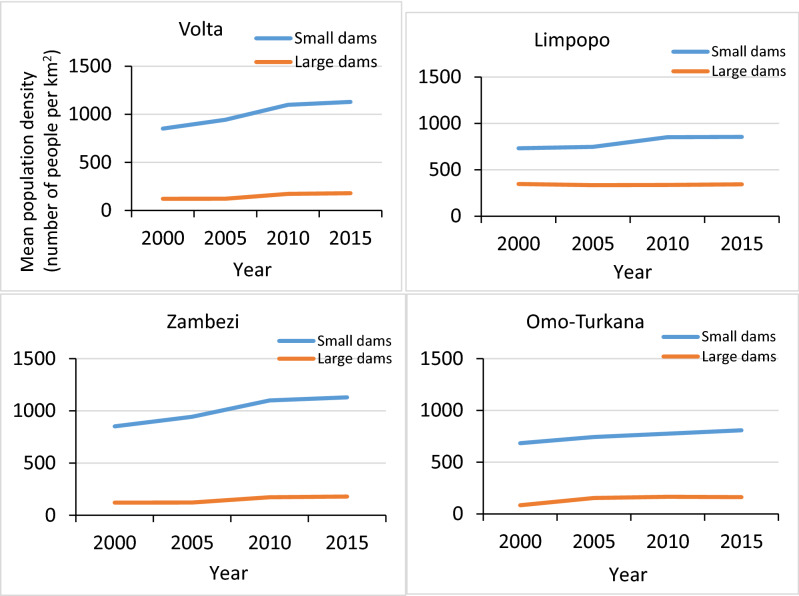
Mean population density (number of people per km2) within the 0–5 km zone of reservoir shoreline in the Volta, Limpopo, Zambezi and Omo-Turkana Basins.

Population (within the 0–5 km cohort) per km of reservoir shoreline was compared between small and large dams in the four basins (Fig. 3). In all four basins, populations increased between 2000 and 2015; on average across all four basins 2.4 × in the vicinity of large dam reservoirs and 1.7 × in the vicinity of small dam reservoirs. Population density was greater in the vicinity of small dams than large dams.

### Malaria incidence: trends in areas of unstable and stable transmission

**Figure 4:**
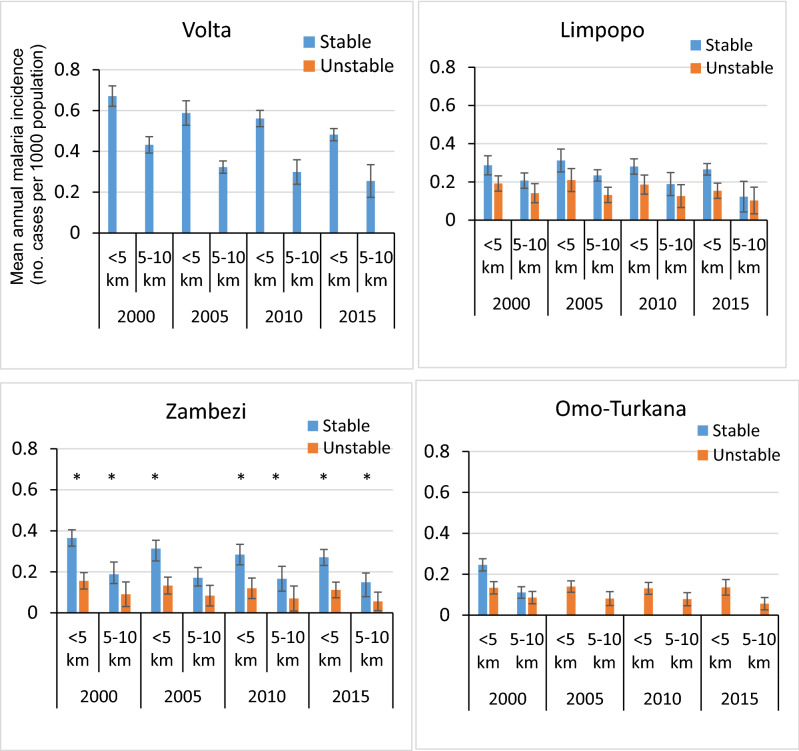
Mean annual malaria incidence in the vicinity of small dams in the Volta, Limpopo, Zambezi and Omo-Turkana Basins. [The error bars indicate standard error of the mean]. [* denotes significant difference in malaria incidence between stable and unstable areas (ANOVA, *P* < 0.05)].

**Figure 5:**
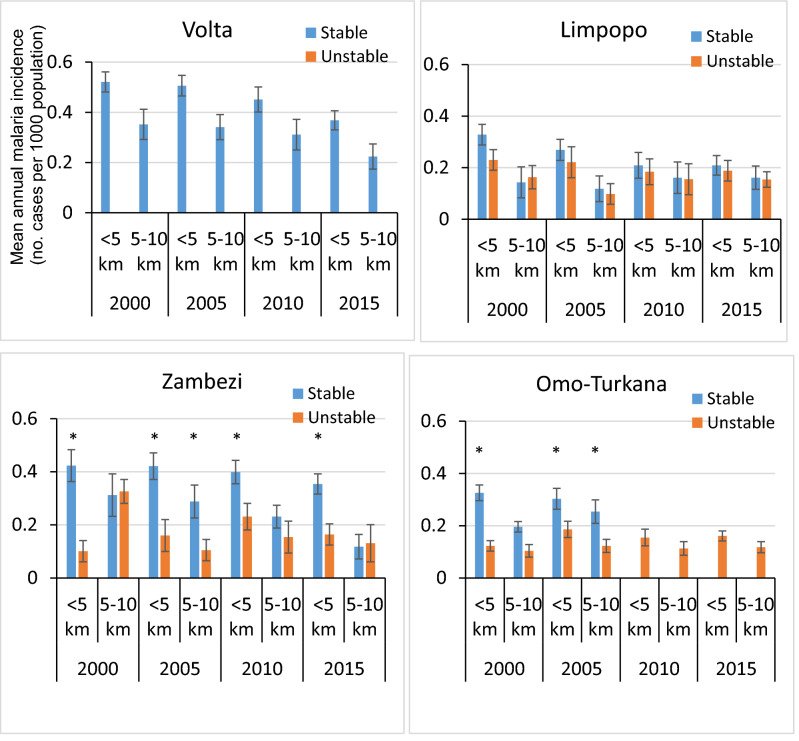
Mean annual malaria incidence in the vicinity of large dam reservoirs in the Volta, Limpopo, Zambezi and Omo-Turkana river basins [The error bars indicate standard error of the mean]. [* denotes significant difference in malaria incidence between stable and unstable areas (ANOVA, *P* < 0.05)].

Annual malaria incidence was generally (though not always) higher in communities living < 5 km from a small dam reservoir than it was for those living 5–10 km from a small dam reservoir (F = 11.341; df = 1; *P* < 0.001) (Fig. 4). Similar results were found for large dams (*P* < 0.001) (Fig. 5). Between 2000 and 2015, the annual malaria incidence showed a broadly declining trend (ANOVA: *P* < 0.001) for both large and small dam reservoirs in areas of stable transmission in all four basins. Similarly, in areas of unstable transmission there were downward trends in malaria transmission in all river basins around both large and small reservoirs.

### Annual malaria cases around large vs. small dams

A total of between 0.87 million and 1.68 million malaria cases were attributable to dams (both small and large) in the four basins depending on the year (Table 4). Of these, 68–89% occurred in areas of stable transmission. Between large and small dams in the present study, the majority of malaria cases in the stable (84–93%) and unstable (71–75%) areas were attributed to the presence of small dams depending on the year. Between 2000 and 2015, more malaria cases could be attributed to dams in the Volta basin than the other three basins combined. Generally, while annual malaria cases attributable to small dams decreased slightly between 2000 and 2015, annual malaria cases attributable to large dams almost doubled over the same period.

**Table 4:** Annual number of malaria cases attributable to small and large dams in Volta, Limpopo, Zambezi and Omo-Turkana Basins in Africa.

	Annual number of malaria cases attributable to dams							
	2000		2005		2010		2015	
	Small dams	Large dams	Small dams	Large dams	Small dams	Large dams	Small dams	Large dams
**Volta**								
Stable	818,535	92,400	678,535	105,571	1,000,658	141,235	83,352	224,187
Unstable	–	–	–	–	–	–	–	–
**Limpopo**								
Stable	140,914	17,332	129,699	16,597	117,861	10,356	60,668	13,793
Unstable	12,275	9089	13,344	10,680	7608	6915	9248	11,039
**Zambez**i								
Stable	446,108	1348	209,392	4285	226,143	6708	242,372	5793
Unstable	124,453	4187	164,862	6613	127,273	12,712	195,498	18,706
**Omo-Turkana**								
Stable	1899	1594		1137	–	–	–	–
Unstable	5846	39,807	8846	42,878	13,621	30,334	5270	56,033
TOTAL	1,550,030	165,757	1,204,678	187,761	1,493,164	208,260	596,408	329,551

#### Shoreline length as malaria incidence determinant

**Figure 6:**
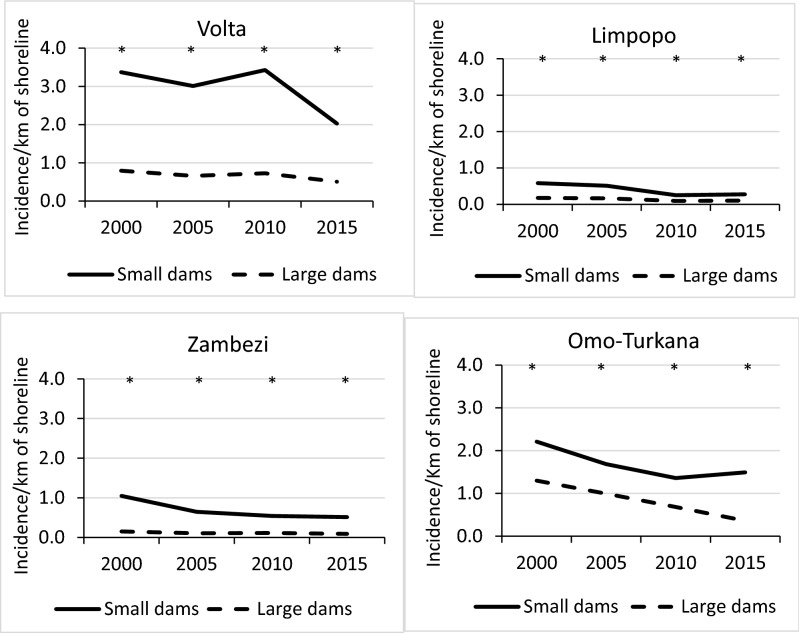
Annual malaria incidence per km of reservoir shoreline in the Volta, Limpopo, Zambezi and Omo-Turkana river basins. [* shows that the difference in malaria incidence/km between dams located in stable and unstable areas for that year was significant (ANOVA; *P* < 0.05)].

In all basins, annual malaria incidence per km of reservoir shoreline was higher for small dams than large dams (*P* < 0.005) (Fig. 6). Between 2000 and 2015, annual malaria incidence per km of shoreline declined in all basins (ANOVA; *P* < 0.01). Small dams in the Volta Basin showed the highest malaria incidence per km of shoreline length (3.5 cases/year per km of shoreline; F = 24.982; df = 3; *P* < 0.001) whereas the lowest values were noted in the Limpopo (less than 0.6 cases/year per km of shoreline). Furthermore, incidence per km was typically 2–7 times greater for small dams than large dams. Overall, small dams represent a greater risk of malaria than large dams in the four basins.

## Discussion

This is the first study to systematically examine the adverse malaria impacts of small dams and to compare those impacts with those arising from large dams. The results highlight two major findings. First, adverse malaria impacts of small dams greatly exceed those of large dams in areas of both stable and unstable transmission. Second, impacts of dams on malaria incidence remained pervasive while population density increased in all four study basins over a period of 15 years. Nevertheless, both large and small dams in the four basins studied continue to exacerbate malaria burden in the vicinity of the reservoirs that they created.

### Significance of small dams

The first major finding suggests that past efforts to approximate the aggregated malaria impacts of water storage infrastructure across SSA—which to date have focused primarily on large dams—are likely to have drastically underestimated the total impact of all dams. The results indicated that between 2000 and 2015 a total of between 0.9 million and 1.7 million malaria cases were attributable to dams in the Limpopo, Omo-Turkana, Volta and, Zambezi basins in SSA. Overall 77–85% of these cases were due to small dams. In western Kenya, McCann et al.^30^ showed that microdam impoundments clearly provided habitat for the malaria vector *An. arabiensis* in the rainy season, most of which was within the shallow apron side of the impoundments where people brought cattle for watering, resulting in compacted soil with aggregations of water-filled hoof prints. This observation suggests a potential conflict between public health concerns about malaria and people’s need for stable and reliable sources of water.

### Explaining small dam impacts

The impact of small dams exceeded the impact of large dams both in absolute terms and also in relation to incidence per kilometre of shoreline. This is likely because populations and population densities are greater in the vicinity of small dams, and because small dam reservoirs tend to provide a more conducive environment for malaria transmission. The small dams, and populations around them, outnumber the large dams in all the four basins, mainly due to their relatively lower construction cost. Most small dams are built for small-scale irrigation and livestock watering and so have to be located close to communities. In contrast, benefits of large dams can be realized great distances from the actual dam and often there is little incentive for elevated population density in the dam vicinity. Hence, much higher absolute numbers and greater population densities are found in the vicinity of small dam reservoirs than in the vicinity of large dam reservoirs.

### Potential influence of an environmental variable: slope

Furthermore, the shoreline slope may be lower around small dams compared to large dams because most large dams are built for hydropower which requires high head. Gentle slope generally corresponds to poor drainage, thereby promoting persistence of surface water bodies and the formation of stable pools convenient for mosquito breeding^29^. In contrast, steeper slope facilitates drainage and reduces the likelihood that pools will form for periods of sufficient duration for mosquitoes to complete their aquatic stages. Slope has been shown as an important topographic factor in determining malaria risk around dams^29^.

### Pervasiveness of malaria around dams

Temporal trends in mean malaria incidence per km of reservoir shoreline showed a declining trend in the vicinity of both large and small dams over the study period, in all four basins (Fig. 6). These downward trends in malaria incidence concur with the broader evidence that unprecedented levels of intervention have resulted in significant progress in most malarious regions of Africa over the past two decades^31^. Nevertheless, the elevated malaria incidence in communities living closer to both small and large dam reservoirs, relative to those located further away, supports the contention that these human-made water bodies continue to constitute hotspots of malaria transmission, even as overall incidence declines. For instance, the humid climate supports stable malaria transmission in the Volta Basin and the construction of Lake Volta, the largest artificial reservoir in the world based on surface area, has been blamed for creating breeding habitats for major malaria mosquitoes in the region^32,33^. Availability of surface water coupled with humid climate and high density of people (Fig. 3) and efficient malaria vectors—*An. gambiae, An. funestus* and *An. arabiensis* (Fig. 2) supported sustained malaria transmission throughout the year. In contrast, much of the Limpopo and the Omo-Turkana basins were unfavourable for major malaria vector mosquitoes due to either aridity (i.e. Omo-Turkana) or the temperate nature of the climate (Limpopo)^34^.

### Implication

A total of 14.8 million people live close to the dams in the four study basins, of which 79–81% live in malarious areas. This is a cause for concern and calls for intensified control efforts. As Africa endeavours to eliminate malaria, such distinct foci of malaria transmission may pose a challenge as they will remain to be residuals for the disease as the map of malaria distribution shrinks. What is also worrisome is that across SSA at least 160 more large dams and many more small dams are currently under construction^35^. And even more are planned as Africa’s climate and development challenges intensify, requiring renewed funding support from donors such as the World Bank. Many of these dams will be located in malarious areas with significant potential to exacerbate malaria burden in local populations.

### Limitations

This study only focussed on four river basins. Other river basins in SSA were not included. Georeferenced data were not available for some dams in the basin, and these dams were excluded from the analyses. The role of environmental and topographic factors on malaria transmission in small vs large dams was not assessed in this study. Future research should investigate the entomological dynamics associated with increased malaria around small dams.

## Conclusions

In recent years, intensified control efforts, catalysed by the Roll Back Malaria initiative, have significantly reduced malaria prevalence and incidence across much of SSA. Against this background, this study has shown that both small and large dams stand-out as hotspots of elevated disease burden. The results also show that much greater malaria impact can be attributed to small dams than large dams, in part because small dams are more abundant and cumulatively far more people live close to the reservoirs that they create. In addition, it is surmised, that environmental factors associated with small dams are more likely to promote transmission.

While there is recognition of dam-malaria linkages, coupling impacts of small and large dams together helps to reveal a larger effect of surface water “storage” on malaria transmission than previously recognized. Concerted effort is needed to address this challenge. Strengthening integrated malaria control efforts in the vicinity of both small and large dam reservoirs including, where technically possible, the use of dam operation as an innovative form of environmental control, should be a critical component of future malaria control efforts. Such a focus is a prerequisite for future strategies to eliminate malaria from SSA to be successful.

## Supplementary Information

Supplementary Information.

## References

[1] African Union. Programme for Infrastructure Development in Africa. Addis Ababa (2015).

[2] Sadoff CW, Hall JW, Grey D, Aerts J, Ait-Kadi M, Brown C (2015). Securing Water, Sustaining Growth: Report of the GWP/OECD Task Force on Water Security and Sustainable Growth.

[3] Kibret S, Wilson GG, Ryder D, Tekie H, Petros B (2017). The influence of dams on malaria transmission in sub-Saharan Africa. EcoHealth.

[4] Atangana S, Foumbi J, Charlois M, Ambroise-Thomas P, Ripert C (1979). Epidemiological study of onchocerciasis and malaria in Bamendjin dam area (Cameroon). Med. Trop..

[5] Oomen J. Monitoring health in African dams: The Kamburu dam as a test case. PhD thesis; Rotterdam University (1981).

[6] King C. The incorporation of health concerns into African River Basin Planning. MIT PhD thesis, Massachusetts Institute of Technology, Cambridge (1996).

[7] Mbakop LR, Awono-Ambene PH, Mandeng SE, Ekoko WE, Fesuh BN, Antonio-Nkondjio C (2019). Malaria Transmission around the Memve’ele Hydroelectric Dam in South Cameroon: A Combined Retrospective and Prospective Study, 2000–2016. Int. J. Environ. Res. Public Health..

[8] Keiser J, Castro MC, Maltese MF, Bos R, Tanner M, Singer BH (2005). Effect of irrigation and large dams on the burden of malaria on a global and regional scale. Am. J. Trop. Med. Hyg..

[9] Lautze J, McCartney M, Kirshen P, Olana D, Jayasinghe G, Spielman A (2007). Effect of a large dam on malaria risk: The Koka Reservoir in Ethiopia. Trop. Med. Int. Health.

[10] Kibret S, Lautze J, Boelee E, McCartney M (2012). How does an Ethiopian dam increase malaria? Entomological determinants around the Koka Reservoir. Trop. Med. Int. Health.

[11] Kibret S, Wilson GG, Ryder D, Tekie H, Petros B (2017). Malaria impact of large dams at different eco-epidemiological settings in Ethiopia. Trop. Med. Int. Health.

[12] Kibret S, Lautze J, McCartney M, Wilson G, Nhamo L (2015). Malaria impact of large dams in sub-Saharan Africa: Maps, estimates and predictions. Malar. J..

[13] Ghebreyesus TA, Haile M, Witten KH, Getachew A, Yohannes AM, Yohannes M, Teklehaimanot HD, Lindsay SW, Byass P (1999). Incidence of malaria among children living near dams in northern Ethiopia: community based incidence survey. BMJ.

[14] Carter RC, Brook JM, Jewsbury JM (1990). Assessing the impact of small dams on vector borne disease. Irrig. Drain. Syst..

[15] Global Water Partnership. Limpopo Basin. https://www.gwp.org/en/WACDEP/IMPLEMENTATION/Where/Limpopo/. Accessed 2 Dec 2019.

[16] FAO. Volta Basin. http://www.fao.org/3/W4347E/w4347e0u.htm. Accessed 2 Dec 2019.

[17] FAO. Zambezi River Basin. http://www.fao.org/3/W4347E/w4347e0o.htm. Accessed 3 Dec 2019.

[18] Hodbod J, Stevenson EG, Akall G, Akuja T, Angelei I, Bedasso EA (2019). Social-ecological change in the Omo-Turkana basin: A synthesis of current developments. Ambio.

[19] European Commission’s Joint Research Center (JRC) global surface water datasets https://global-surface-water.appspot.com/. Accessed 12 May 2018.

[20] Food and Agriculture Organization (FAO). African dams. http://www.fao.org/nr/water/aquastat/dams/index.stm. 2010. Accessed 12 May 2018.

[21] International Commission for Large dams (ICOLD). World register of dams. http://www.icold-cigb.org/GB/World_register/world_register.asp (2010). Accessed 15 May 2018.

[22] International Rivers. African dams briefing. http://www.internationalrivers.org/files/attached-files/afrdamsbriefingjune2010.pdf 2010. Accessed 12 May 2018

[23] Malaria Atlas Project. Big Data Institute, University of Oxford. www.map.ox.ac.uk. Accessed 2 June 2018.

[24] Worldpop Project. World population data. (2014). http://www.worldpop.org.uk/data/. Accessed 2 June 2018.

[25] Kauffman C, Briegel H (2004). Flight performance of the malaria vectors *Anopheles gambiae* and *Anopheles atroparvus*. J. Vector Ecol..

[26] Gething PW, Patil AP, Smith DL, Guerra CA, Elyazar IR, Johnston GL (2011). A new world malaria map: *Plasmodium falciparum* endemicity in 2010. Malar. J..

[27] Kiszewski A, Mellinger A, Spielman A, Malaney P, Sachs SE, Sachs J (2004). A global index representing the stability of malaria transmission. Am. J. Trop. Med. Hyg..

[28] Hershberger SL, Moskowitz DS (2014). Modeling Intraindividual Variability with Repeated Measures Data: Methods and Applications.

[29] Kibret S, Lautze J, McCartney M, Nhamo L, Yan G (2019). Malaria around large dams in Africa: Effect of environmental and transmission endemicity factors. Mal. J..

[30] McCann RS, Gimnig JE, Bayoh MN, Ombok M, Walker ED (2018). Microdam impoundments provide suitable habitat for larvae of malaria vectors: An observational study in Western Kenya. J. Med. Entomol..

[31] Bhatt S, Weiss DJ, Cameron E, Bisanzio D, Mappin B, Dalrymple U (2015). The effect of malaria control on *Plasmodium falciparum* in Africa between 2000 and 2015. Nature.

[32] Mba CJ, Aboh IK (2006). Prevalence and management of Malaria in Ghana: A case study of volta region. Afr. Pop. Stud..

[33] Krefis AC, Schwarz NG, Krüger A, Fobil J, Nkrumah B, Acquah S (2011). Modeling the relationship between precipitation and malaria incidence in children from a holoendemic area in Ghana. Am. J. Trop. Med. Hyg..

[34] Mosase E, Ahiablame L (2018). Rainfall and temperature in the Limpopo River Basin, Southern Africa: Means, variations, and trends from 1979 to 2013. Water.

[35] International Rivers. (2015). The World Bank and Dams. https://www.internationalrivers.org/sites/default/files/attached-files/world_bank_and_dams_fact_sheet_web.pdf. Accessed 15 Feb 2019.

