# Supplementary material for: The impact of large and small dams on malaria transmission in four basins in Africa
Source: Sci Rep. 2021 Jun 25;11:13355. doi: 10.1038/s41598-021-92924-3 (PMC8233325; doi:10.1038/s41598-021-92924-3)
Supplement: Supplementary file 1 — Supplementary Information. [file 41598_2021_92924_MOESM1_ESM.docx]

# **The impact of large and small dams on malaria transmission in four basins in Africa**

# Solomon Kibret, Matthew McCartney, Jonathan Lautze, Luxon Nhamo, Guiyun Yan

# **Estimating Reservoir Characteristics**

In this study, it was intended to derive the size, topographical and climate characteristics of reservoirs in Africa at multiple scales. At the entire continental scale, the study looked into the characteristics of large man-made reservoirs. The study specifically estimated:

- Area and perimeter of the reservoir at its maximum extent
- Area and slope characteristics of the seasonally submerged part of these large reservoirs

At basin scale, the study was focused on identifying the small reservoirs in the Limpopo, Zambezi, Omo-Turkana and Volta river basins. Reservoirs with a surface area of less than 100 ha were considered as small reservoirs for the purpose of this study.

## Data

**Global Water Surface Layers**

The European Commission’s Joint Research Center developed global water surface datasets in the framework of the Copernicus Programme.

JRC Yearly Water Classification History v1.0 data set published through Google Earth Engine was used for this study. This dataset contains maps of the location and various aspects of temporal distribution of surface water from 1984 to 2015 at global scale generated from Landsat images with 30m resolution. The Yearly Seasonality Classification collection contains a year-by-year classification of the seasonality of water based on the occurrence values detected throughout the year.

| **Band Name** | **Description** | **Classification** |
| --- | --- | --- |
| waterClass | Classification of the seasonality of water throughout the year. | 0 = no data 1 = not water 2 = seasonal water 3 = permanent water |

We used the data from 2000 to 2015 for this study. The permanent and seasonal areas for each year was combined using Google Earth Engine to derive the annual maximum extent of the water bodies. Those 16 annual maximum water extent layers were combined to derive the 16-year maximum extent of the waterbodies and this was used to extract the perimeter of the reservoirs at its maximum extent.

Permanent water extent of the reservoirs for 16 year time period was the other variable required to calculate the seasonally submerged area once the 16 years maximum extent was available.

The JRC Yearly Water Classification History v1.0 data set has demarcated annual extent of permanent water presence for each year. The 16-year permanent water extent was determined by identifying the common areas across all the annual permanent water extent layers.

The outputs were downloaded as 1 degree tiles from Google Earth Engine and combined to produce the continental scale layers.

**SRTM DEM**

The Shuttle Radar Topography Mission (SRTM) was flown aboard the space shuttle Endeavour February 11-22, 2000. This mission used single-pass interferometry, which acquired two signals at the same time by using two different radar antennas. Differences between the two signals allowed for the calculation of surface elevation. Endeavour orbited Earth 16 times each day during the 11-day mission, completing 176 orbits over 80% of the Earth's land surface between 60° north and 56° south latitude with data points posted every 1 arc-second (approximately 30 meters) (<https://lta.cr.usgs.gov/SRTM1Arc>) and <=16m absolute vertical accuracy. The data is available with a precision up to the nearest meter.


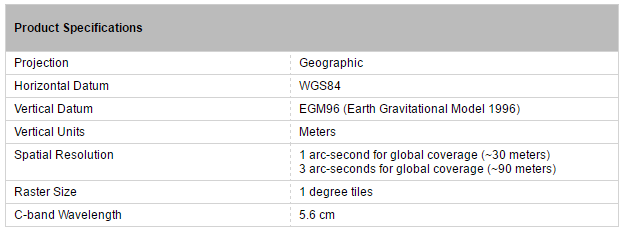


This SRTM 30m digital elevation model was used to extract the altitude of the reservoir and slope (in degrees) derived from DEM used to calculate the mean slope of the seasonally submerged water areas.

# **Topographic data of Large Dams in Africa**

The large dams location data set from International Commission of Large Dams was used as the initial data set for this study. The data set has a list of 1285 dams with geographic coordinates. It was found that the location information has many errors and a large number of points were not located at or near the dams specified. We used several online data sources such Google Earth, FAO AquaStat, Global Reservoirs and Dams (GRanD) database, and many other online maps and location data sets to verify and correct the location.

The overall status are as follows;

| Total Number of points | 1285 |
| --- | --- |
| Correctly located | 956 |
| Duplicate Entries | 42 |
| Incomplete/Proposed/Canceled | 133 |
| Cannot locate | 154 |

The 16 year (2000 – 2015) maximum water surface extent layer described above was used to find the maximum extent of the identified reservoirs. The 16-year permanent water extent layer used to get the areas permanently submerged under water for 16 years.

The difference between the maximum water extent and the permanent water extent was extracted as seasonal submergence areas in ha and the average slope of these regions were estimated from the slope layer. Subsequently altitude (m), mean rainfall (mm) and mean temperature (degree Celsius) extracted respectively from SRTM 30m DEM.

Among correctly located 956 dams, 38 structures did not forma definite reservoir boundary. Some of these may be barrages or weirs which do not form a reservoir and in some cases, the reservoir part is not wider than the incoming river and it was not possible to determine the exact reservoir boundary (Supplementary figure S1). Therefore, all 8 parameters mentioned above were calculated only for remaining 918 dams.

| 16 year maximum water surface extent area | Image from Google Earth |
| --- | --- |
| 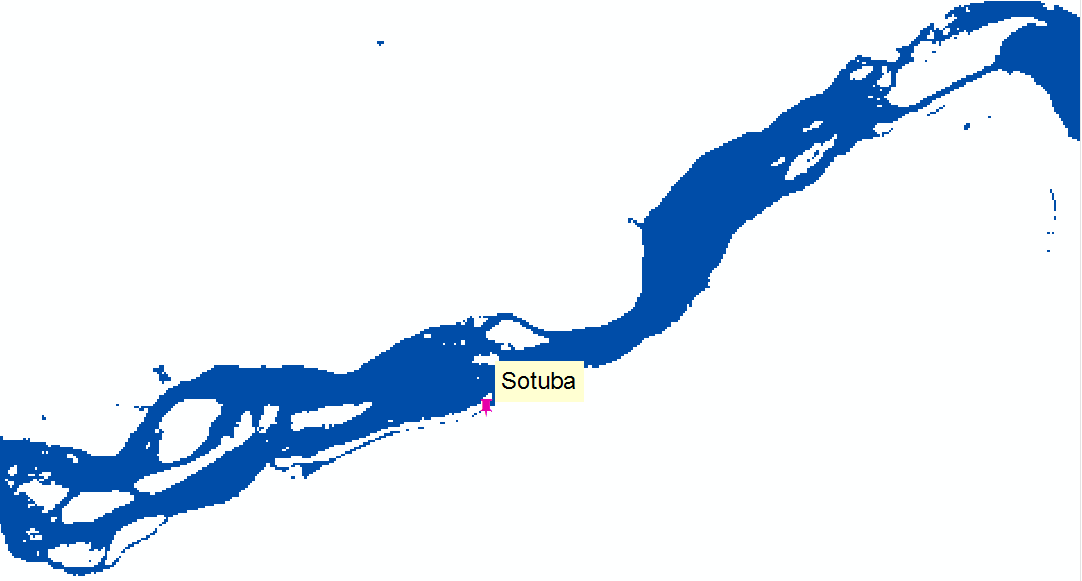 | 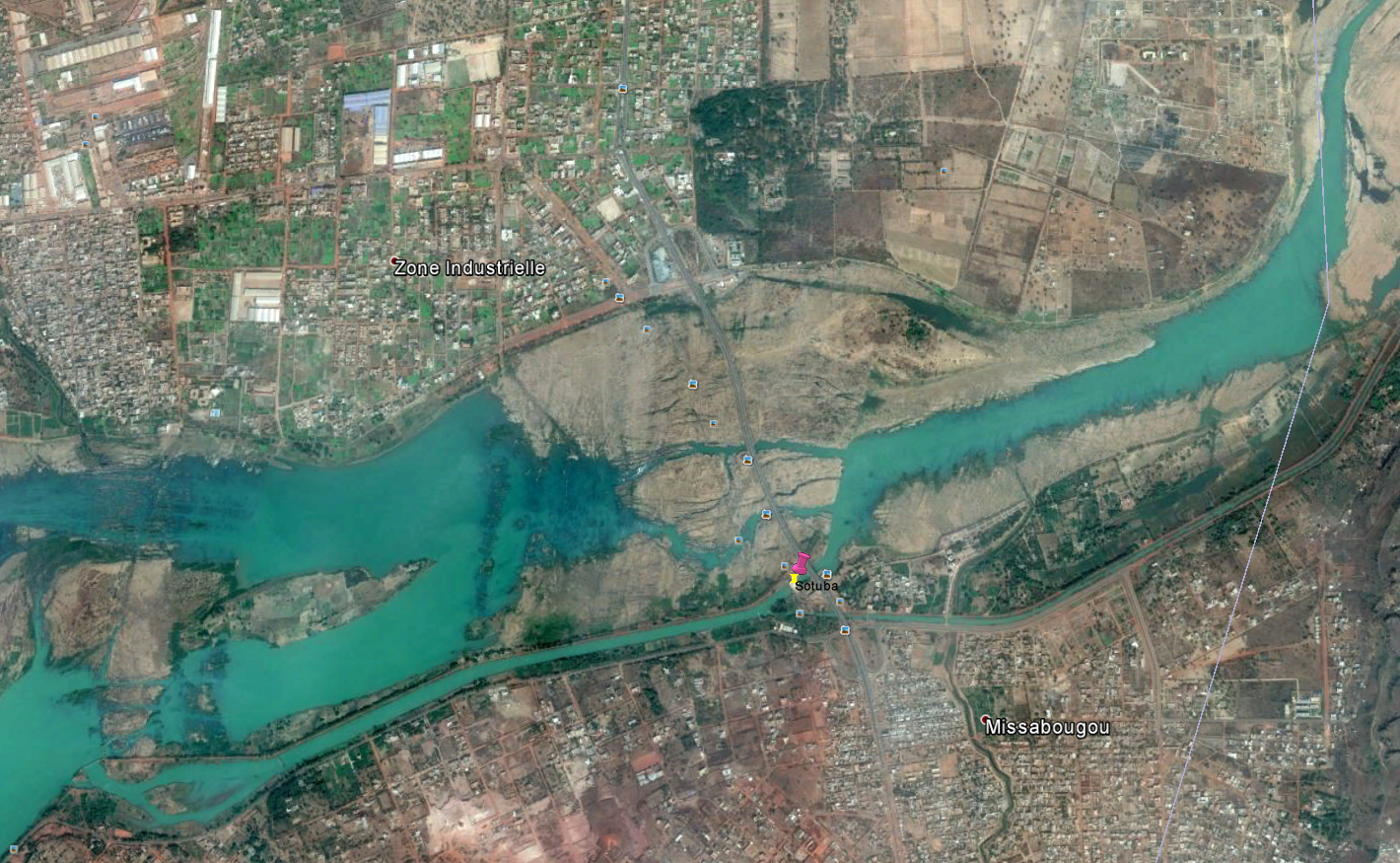 |
| 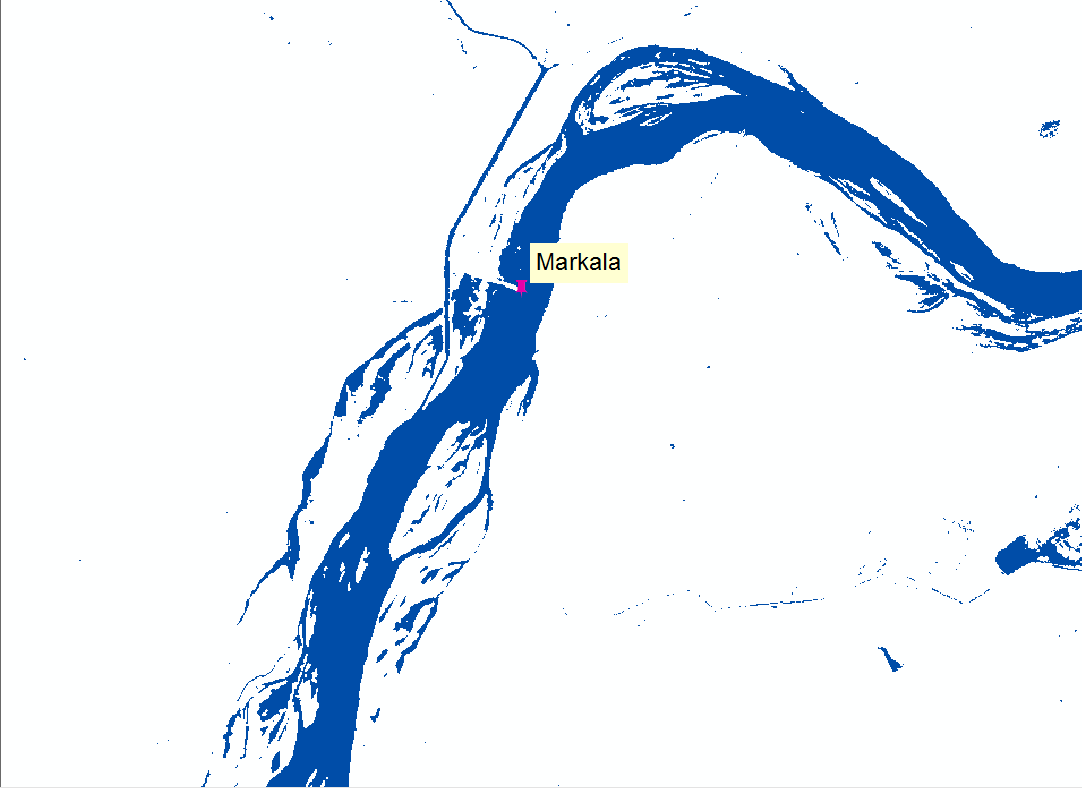 | 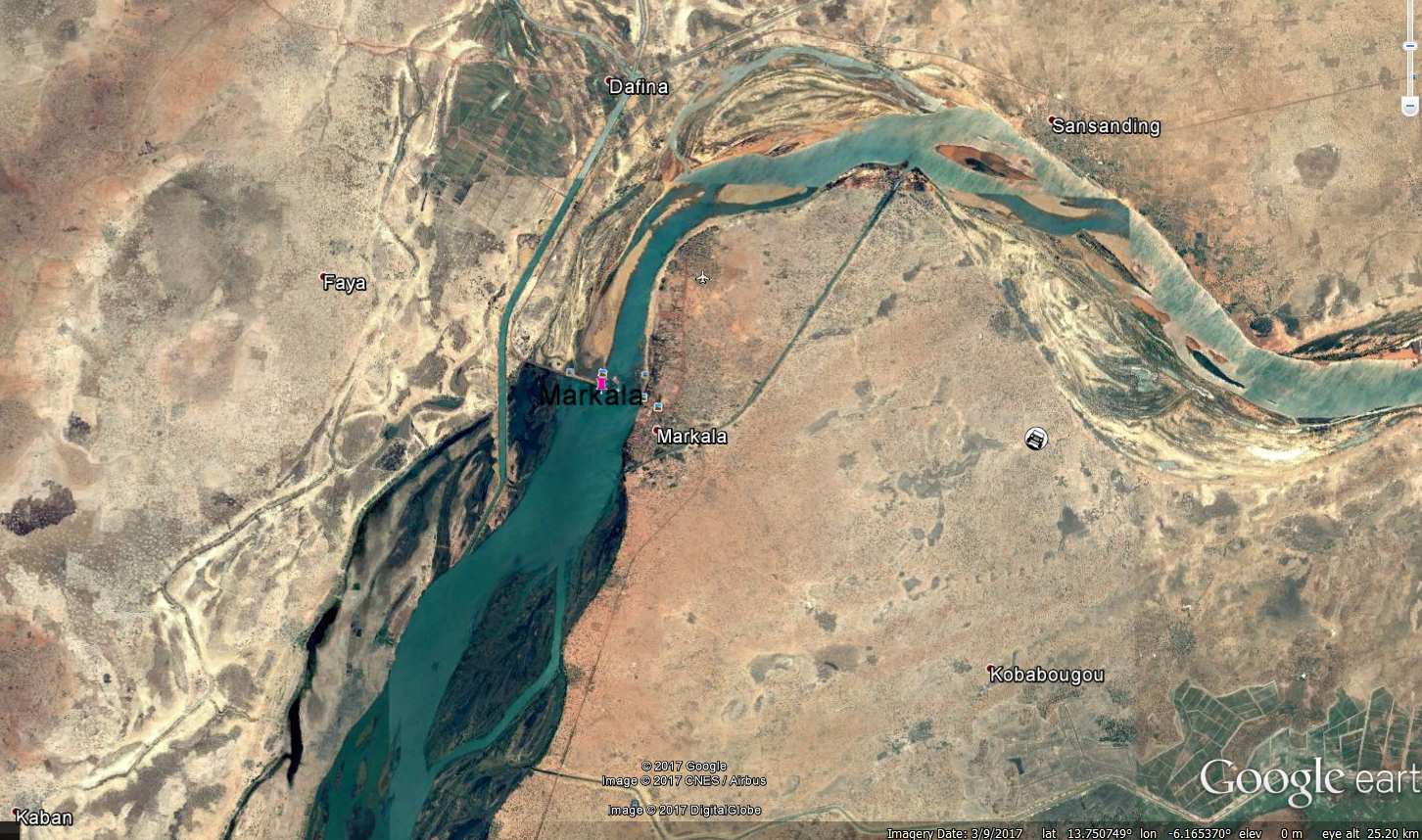 |
| 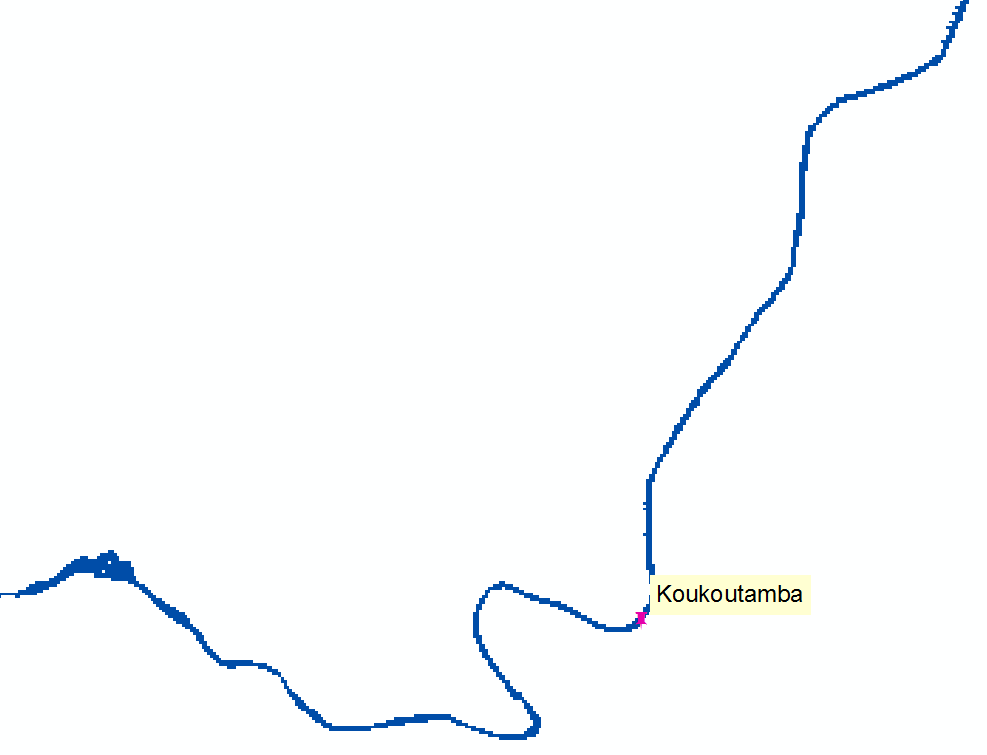 | 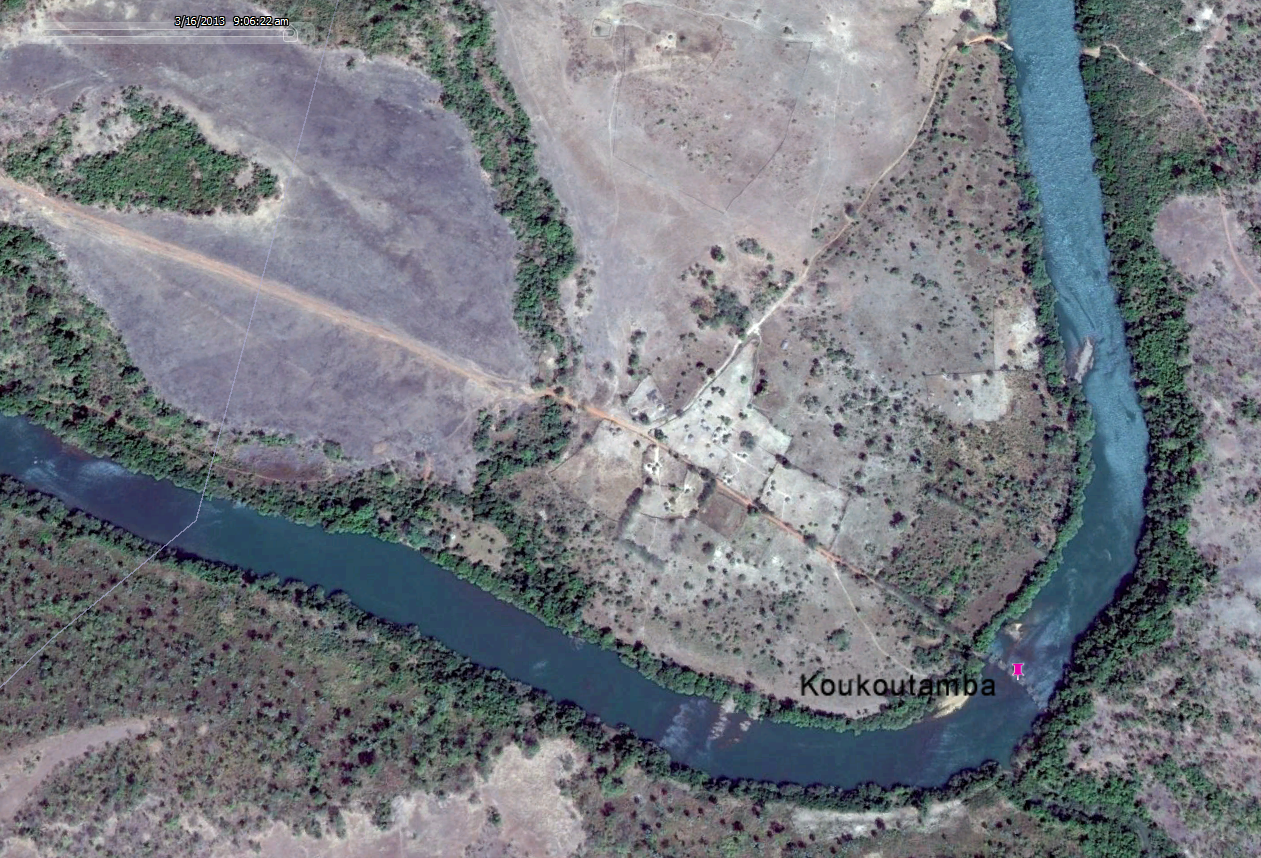 |

**Supplementary figure S1***.* Dams which are not forming definite reservoir boundary based on Google Earth Imagery (©Google, CNES/Airbus, Maxar Technologies)

## **Identification of Small Reservoirs in Volta and Limpopo River Basins**

The reservoirs were identified using the 16-year maximum water surface extent layer derived from the JRC Yearly Water Classification History v1.0 data set.

The layer demarcated a total of 36,959 waterbodies in the Volta Basin. Similarly, 64,162 waterbodies were found in Limpopo basin. The reservoirs less than 100 ha were considered as small reservoirs for the purpose of this study. All the waterbodies with a surface area from 2 ha to 100 ha were checked with Google Earth images to distinguish between reservoirs and natural water bodies. The results are summarized below:

|  | **VOLTA** | **LIMPOPO** | **Omo Turkana** | **Zambesi** |
| --- | --- | --- | --- | --- |
| Total number of water bodies | 36,959 | 64,162 | 51559 | 524534 |
| Total areal extent of water bodies (Km^2^) | 9307.55 | 2724.82 | 2226847.88 | 1380197.466 |
| No of single pixel water bodies | 18,078 | 28,228 | 26823 | 235386 |
| Number of small reservoirs (2-100 ha) | 717 | 1,656 | 39 | 2477 |

Size distribution of Waterbodies

| Area(ha) | No. of Waterbodies in Volta Basin | No. of Waterbodies in Limpopo Basin | No. of Waterbodies in Omo Turkana Basin | No. of Waterbodies in Zambesi Basin |
| --- | --- | --- | --- | --- |
| 2 - 10 | 346 | 1122 | 26 | 1540 |
| 11 - 20 | 172 | 290 | 7 | 456 |
| 21 - 30 | 78 | 112 | 3 | 199 |
| 31 - 40 | 41 | 53 | 1 | 106 |
| 41 - 50 | 23 | 31 | 1 | 56 |
| 51 - 60 | 15 | 23 | 1 | 46 |
| 61 - 70 | 15 | 10 | 0 | 39 |
| 71 - 80 | 8 | 7 | 0 | 17 |
| 81 - 90 | 6 | 6 | 0 | 13 |
| 91 - 100 | 13 | 2 | 0 | 5 |
| Total | 717 | 1656 | 39 | 2477 |

**Estimate monthly water surface area for selected dams in Africa**

## Introduction

The purpose of this study was to derive the monthly water surface area for 449 dams in Africa for year 2000, 2005, 2010 and 2015.

## Data

The European Commission’s Joint Research Center developed global water surface datasets in the framework of the Copernicus Programme.

JRC Monthly Water Classification History v1.0 data set contains maps of the location and temporal distribution of surface water from 1984 to 2015 and published through Google Earth Engine. These data were generated using 3,066,102 scenes from 30m resolution Landsat 5, 7 and 8 acquired between 16 March 1984 and 10 October 2015.

The Monthly History collection holds the entire history of water detection on a month-by-month basis. The collection contains 380 images, one for each month between March 1984 and October 2015.

| **Band Name** | **Description** | **Label** |
| --- | --- | --- |
| water | Water detection | 0 = no data 1 = not water 2 = water |

## Method

The dataset was not available to direct download so had to script in google code engine in order to download. 16 year maximum water extent of reservoir derived from earlier study was used to subset the monthly water surface layer and reservoir vise monthly layers were downloaded. Subsequently monthly mosaiced layer generated and after converting all layers from raster to vector, area calculation has been performed.

But for some months, some reservoirs were partially or fully not covered by the respective monthly surface water layer due to cloud cover or unavailability of Landsat satellite images and “0-no data” value recorded in images to represent those areas. When area of “No data” was less than 30% of maximum surface water area of a reservoir, monthly surface area extracted from the layer otherwise recorded as “No Sufficient Data”.

**Summary of parameters estimated for large and small reservoirs**

| Parameters | large reservoirs with dams | large dams with no reservoir | Small reservoirs in Limpopo | Small reservoirs in Volta |
| --- | --- | --- | --- | --- |
| Maximum reservoir area (ha) | ✓ | X | ✓ | ✓ |
| Maximum perimeter (km) | ✓ | X | ✓ | ✓ |
| Seasonally Submerged area (ha) | ✓ | X | X | X |
| Mean slope (deg) | ✓ | X | X | X |
